# Thoracic Purpura in a Patient with Myelodysplastic Syndrome

**DOI:** 10.5826/dpc.1104a113

**Published:** 2021-10-01

**Authors:** Nuno Gomes, Filomena Azevedo, Inês Brito, Cármen Lisboa

**Affiliations:** 1 Centro Hospitalar Universitário de São João, Dermatovenereology Department, Porto, Portugal; 2 Centro Hospitalar Universitário de São João, Clinical Hematology Department, Porto, Portugal; 3 Microbiology Division of Pathology Department and Center for Health Technology and Services Research (CINTESIS), Faculty of Medicine, University of Porto, Portugal

## Case Presentation

**Figure 1:**
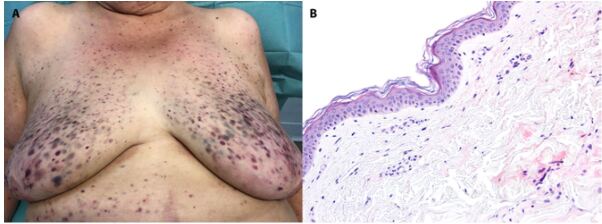
(A) Clinical presentation with several erythematoviolaceous macules on the thorax. (B) Skin biopsy showing a mild superficial perivascular dermatitis with rare lymphocytes and a thin epidermis (H&E, ×200).

A 73-year-old woman with pulmonary hypertension and high-risk myelodysplastic syndrome (MDS) developed asymptomatic erythematous-violaceous macules on her thorax (Figure 1A). Macules evolved for 2 weeks, while she was being treated with prednisolone for an auto-immune hemolytic anemia. The differential diagnosis included drug or infectious-induced vasculitis, and MDS-related autoimmune phenomena. Complete blood count revealed low hemoglobin (11.1 g/dL, normal >12.0), low white cells (2.5*10^9^/L, normal>4*10^9^/L), and low platelets (64*10^9^/L, normal>150*10^9^/L). The coagulation study and auto-immune markers were normal. Skin biopsy showed mild perivascular dermatitis (Figure 1B) and negative direct immunofluorescence, consistent with vasculitis-induced purpura confined to the thorax. Due to MDS progression, the patient started azacytidine 1 month after the onset of dermatosis. After 2 cycles, she developed a sudden episode of hemoptysis and died, with skin lesions overlapping the initial case presentation.

## Teaching Point

MDS is frequently associated with autoimmune disorders, which may worsen survival rates [1,2]. In this case, the combination of MDS and pulmonary hypertension resulted in an impressive clinical presentation with sudden unfortunate outcome.
